# LinChemIn: SynGraph—a data model and a toolkit to analyze and compare synthetic routes

**DOI:** 10.1186/s13321-023-00714-y

**Published:** 2023-04-01

**Authors:** Marta Pasquini, Marco Stenta

**Affiliations:** Syngenta Crop Protection AG, Schaffhauserstrasse, 4332 Stein, AG Switzerland

**Keywords:** Reaction, Chemoinformatics, Computer-aided synthesis planning

## Abstract

**Background:**

The increasing amount of chemical reaction data makes traditional ways to navigate its *corpus* less effective, while the demand for novel approaches and instruments is rising. Recent data science and machine learning techniques support the development of new ways to extract value from the available reaction data. On the one side, Computer-Aided Synthesis Planning tools can predict synthetic routes in a model-driven approach; on the other side, experimental routes can be extracted from the Network of Organic Chemistry, in which reaction data are linked in a network. In this context, the need to combine, compare and analyze synthetic routes generated by different sources arises naturally.

**Results:**

Here we present LinChemIn, a python toolkit that allows chemoinformatics operations on synthetic routes and reaction networks. Wrapping some third-party packages for handling graph arithmetic and chemoinformatics and implementing new data models and functionalities, LinChemIn allows the interconversion between data formats and data models and enables route-level analysis and operations, including route comparison and descriptors calculation. Object-Oriented Design principles inspire the software architecture, and the modules are structured to maximize code reusability and support code testing and refactoring. The code structure should facilitate external contributions, thus encouraging open and collaborative software development.

**Conclusions:**

The current version of LinChemIn allows users to combine synthetic routes generated from various tools and analyze them, and constitutes an open and extensible framework capable of incorporating contributions from the community and fostering scientific discussion. Our roadmap envisages the development of sophisticated metrics for routes evaluation, a multi-parameter scoring system, and the implementation of an entire “ecosystem” of functionalities operating on synthetic routes. LinChemIn is freely available at https://github.com/syngenta/linchemin.

**Graphical Abstract:**

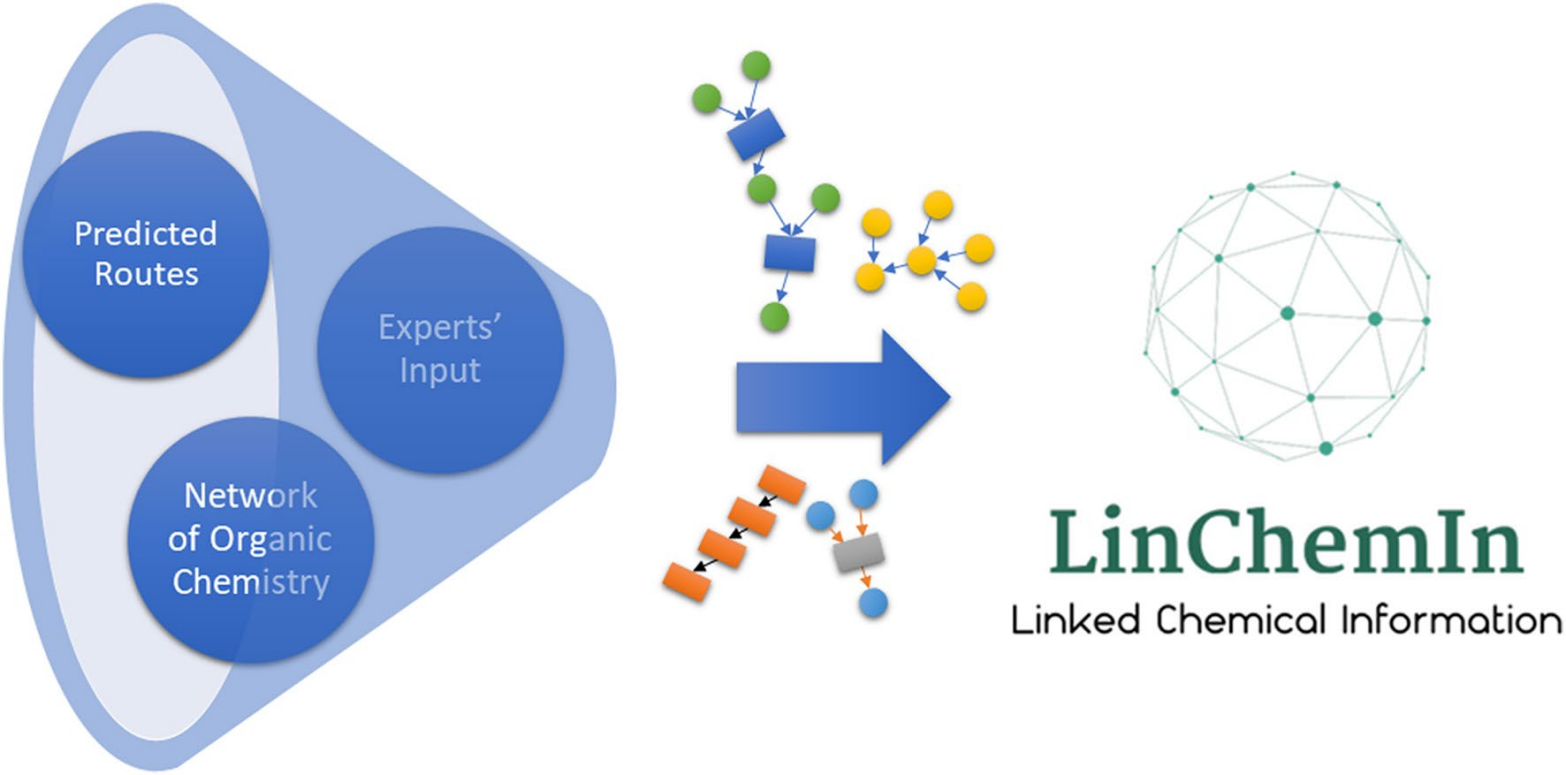

**Supplementary Information:**

The online version contains supplementary material available at 10.1186/s13321-023-00714-y.

## Introduction

Chemists are paying a renewed interest in chemical reaction data [1, 2] and seek novel ways to extract value from them. The accurate prediction of retrosynthetic routes [3, 4], the analysis [5] and optimization [6, 7] of existing reaction classes, or the discovery [8–11] of new ones are just few examples that highlight the impact of reaction analytics and reactivity modeling on chemical science. Such fundamental shift in how scientists consume and use this data creates a demand for innovative ways to shape raw data points, excerpt insightful information, and build actionable knowledge. Moreover, on the one side, the traditional combination of visual inspection and databases searches is made progressively less effective by the increased amount of available reaction data. On the other side, some innovations in data science and machine learning provide more efficient instruments to navigate the available information to support scientists in making data-informed decisions faster.

CASP (Computer-Aided Synthesis Planning) [3, 12–18] tools constitute model-driven approaches to navigating the *corpus* of reaction data and have proven to be able to predict retrosynthetic routes even for completely new compounds. The usage of these tools can be considered complementary to the conventional construction of synthetic procedures via iterative database searches. The experimental reaction data available from the literature, from electronic lab notebooks, etc., as well as computationally generated reaction data [19, 20], can be arranged in a network in which sequences of chemical reactions are linked through common molecular intermediates, generating the so-called Network of Organic Chemistry (NOC) [8, 21]. The NOC is often instantiated as graph databases to leverage graph arithmetic [16] for unconventional search procedures, such as extracting chemical routes [22–25]. This arrangement of the data allows scientists to efficiently navigate the preexisting knowledge and to enrich it by identifying new potential routes for known target compounds.

From these premises, a natural need arises to combine the routes predicted from CASP tools with those extracted from NOC, to perform comparison, validation, consensus, and diversity analyses across different sources and methods.

Synthetic chemists seek a better balance between performance and sustainability in chemical processes [26–28]. Synthetic biologists and systems chemists analyze biotic reaction networks [29, 30] to understand or design new bio-inspired reactive systems [31–38]. Scientists in these disciplines need a representation of chemical reaction data that explicitly accounts for the connectivity between chemical reactions. Moreover, with the emphasis shifting from individual reactions to reaction sequences, scientists’ requirements increase accordingly, creating a pressing demand for route-level analytic based on quantitative descriptors and multi-parameter metrics. In addition to a general data model, they need tools (software, databases, etc.) to manipulate and investigate sequences and networks of chemical reactions.

While only a few tools are publicly available for querying NOC databases [22, 25, 39], the number of available CASP tools is rapidly increasing [3, 4, 39–41], including those accounting for bio-catalyzed reactions [36]. Whether predicted or extracted, synthetic routes come in different formats, often holding mutually incompatible data models, depending on the source (NOC/CASP) and origin (specific CASP platform or tool). Given the rapid evolution of the field and the growing number of method developers, imposing any unified standard of format and data model across applications and platforms appears daunting. As a lightweight alternative, we envision bespoke software that operates translations between data formats and conversion between data models, allowing third-party applications to consume the output of multiple NOC and CASP tools.

To fill this gap in the chemoinformatics landscape, we present a chemical expert system that will take for chemical routes and networks the role that toolkits like OpenBabel, RDKit, and CDK play for molecules. LinChemIn captures the Linked Chemical Information that underlies chemical reaction data and allows managing entities of chemical relevance, such as the synthetic chemical route. Besides the interconversion of file formats and data models, the toolkit enables route-level analysis and operations, including network editing, descriptor calculations, topological and chemical route comparisons, and searches. On the one hand, application scientists can conveniently access the toolkit functionalities via command-line interfaces (CLIs) and front-facing high-level software components. On the other hand, developers of third-party applications can access lower-level modules to obtain complete control of the toolkit functionalities. Besides these two user categories, we designed the toolkit architecture to encourage scientists and developers to contribute to its code base. The first release of LinChemIn constitutes an open and extensible framework capable of incorporating contributions from the scientific community. Following the AGILE approach to software development, the early publication of a minimal viable product should stimulate scientific discussions and provide valuable steers to further improvements. While pursuing an active development roadmap, we will progressively release new modules that enable new functionalities.

This introductory article describes the LinChemIn toolkit and its benefits to different potential user categories. First, we report the domain analysis, where a formal representation of the domain knowledge leads to a series of scientific and technical requirements. Next, we provide some implementation details, discussing our software architecture choices while translating the scientific requirements into computational procedures. Then, we present some applicative examples to demonstrate the functionalities implemented in the toolkit and discuss the outcome of some illustrative analyses. After that, we exemplify how developers could extend the code to incorporate new functionalities. Finally, we offer a glimpse of the LinChemIn development roadmap, highlighting the most significant functionalities planned for future releases.

## Domain modeling

**Fig. 1:**
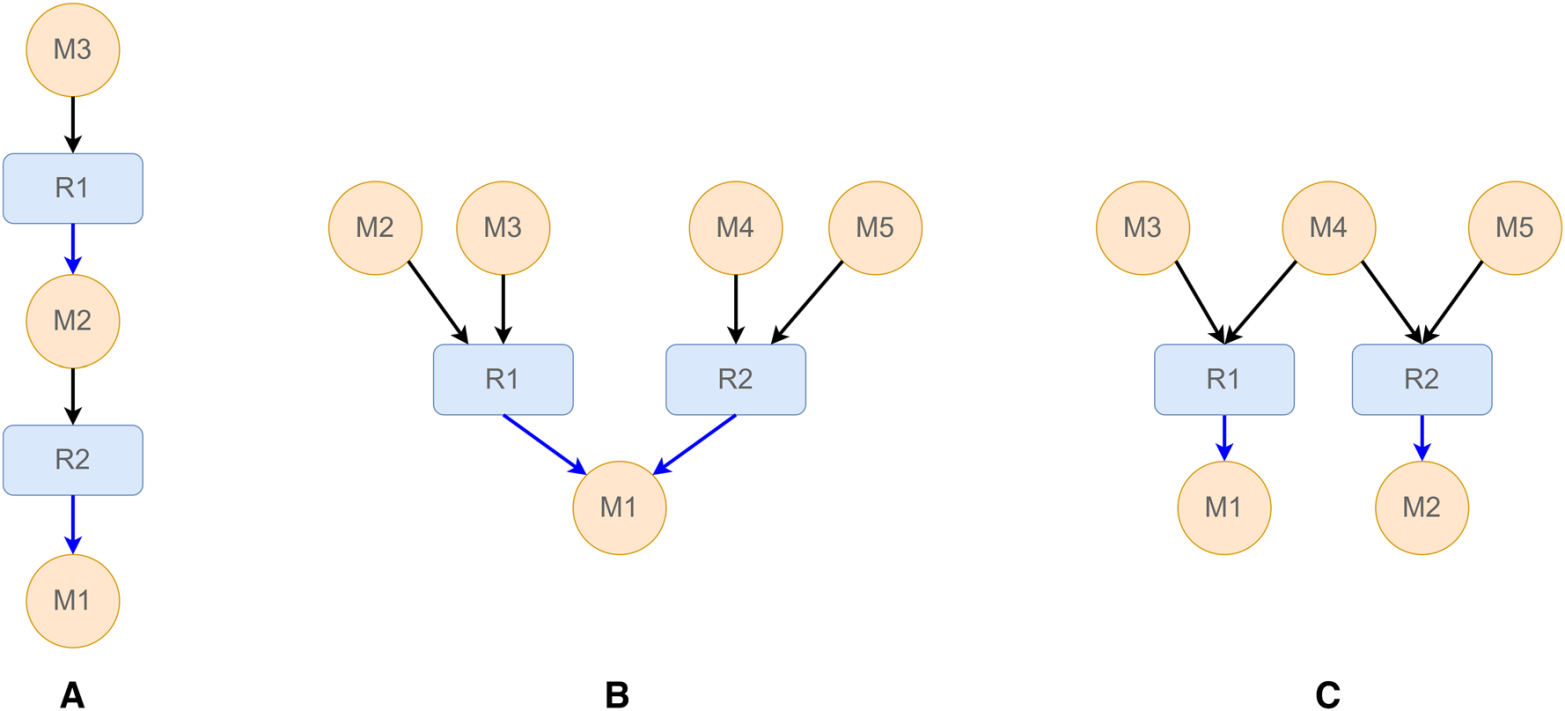
Types of connections between reactions. **A** M2 is a molecular intermediate produced by reaction R1 and consumed by reaction R2. **B** M1 is a chemical produced by both R1 and R2, and represents a convergence point. **C** M4 is a chemical acting as reactant for both R1 and R2, and represents a divergence point

In our development work, we adopt Domain-Driven Design (DDD) principles to ensure a close match between the scientific domain (in this case, synthetic chemistry) and the structure and language of the LinChemIn software code. This approach fosters constructive collaboration between technical and domain experts by placing the project’s primary focus on domain logic. The domain modeling of the LinChemIn project captures the relationships between distinct chemical reactions, whose most common cases are represented in Fig. 1. Intermediates are chemicals produced by one reaction and consumed by another (Fig. 1A, M2). The chemical shared as a product by two or more reactions (Fig. 1B, M1) is a convergence point, indicating alternative ways to deliver a compound. The chemical shared as a reactant by two or more reactions (Fig. 1C, M4) is a common intermediate, a divergence point. Together these reaction links define the Network of Organic Chemistry, described in detail in several works [8, 21].

This section describes the data models we created to map a subset of the concepts relevant to chemical synthesis. We build a list of operations necessary to map the business logic of the scientific domain into algorithms and software.

**Fig. 2:**
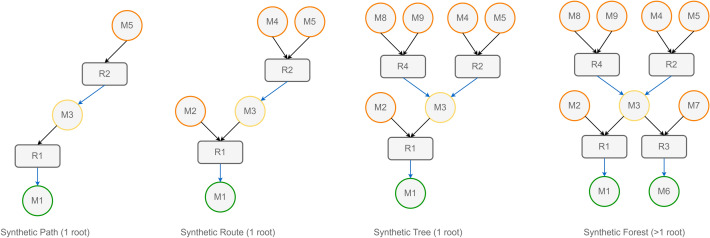
Schematic representation of different SynGraph types. Synthetic Path: a linear sequence connecting a root and a leaf. Synthetic Route: the union of Synthetic Paths starting from a single root that is collectively necessary and sufficient to represent a chemical synthesis. Synthetic Tree: the union of Synthetic Routes sharing the same root. Synthetic Forest: the union of Synthetic Trees with distinct roots

The most granular concepts we encounter are those of chemical compounds and chemical reactions, mapped as Molecule and Chemical Equation^1^. We select a set of immutable properties that define their identity so that we can treat them as *value objects*. In the case of Molecule entities, we use an attribute derived from the molecular structure like SMILES or the InChiKey strings. If we formalize chemical reactions as chemical compounds that have a role (reactants, products, reagents), the set of Molecule entities and a reaction role map suffice to define the nature of a Chemical Equation. As *value objects*, Molecule and Chemical Equation instances constitute the nodes of a graph representation of the reaction network. The SynGraph (synthetic graph) is the central data object in LinChemIn. It contains Chemical Equations and Molecules alongside their relationships. Relevant scientific concepts map onto particular SynGraph sub-types, as shown in Fig. 2. For instance, we define the synthetic route as a set of unique (mutually exclusive) chemical reaction steps that, arranged into a (possibly branched) sequence, are necessary and sufficient (collectively exhaustive) to assemble a target compound from starting material. We distinguish the synthetic route from simple synthetic paths, connecting the target compound to a starting material through a linear sequence. In addition, we envision aggregative data models such as the synthetic tree (collection of synthetic routes) and the synthetic forest (collection of synthetic trees), which are necessary to collect and compare routes from different sources (CASP, NOC, etc.) for one or more targets.

**Fig. 3:**
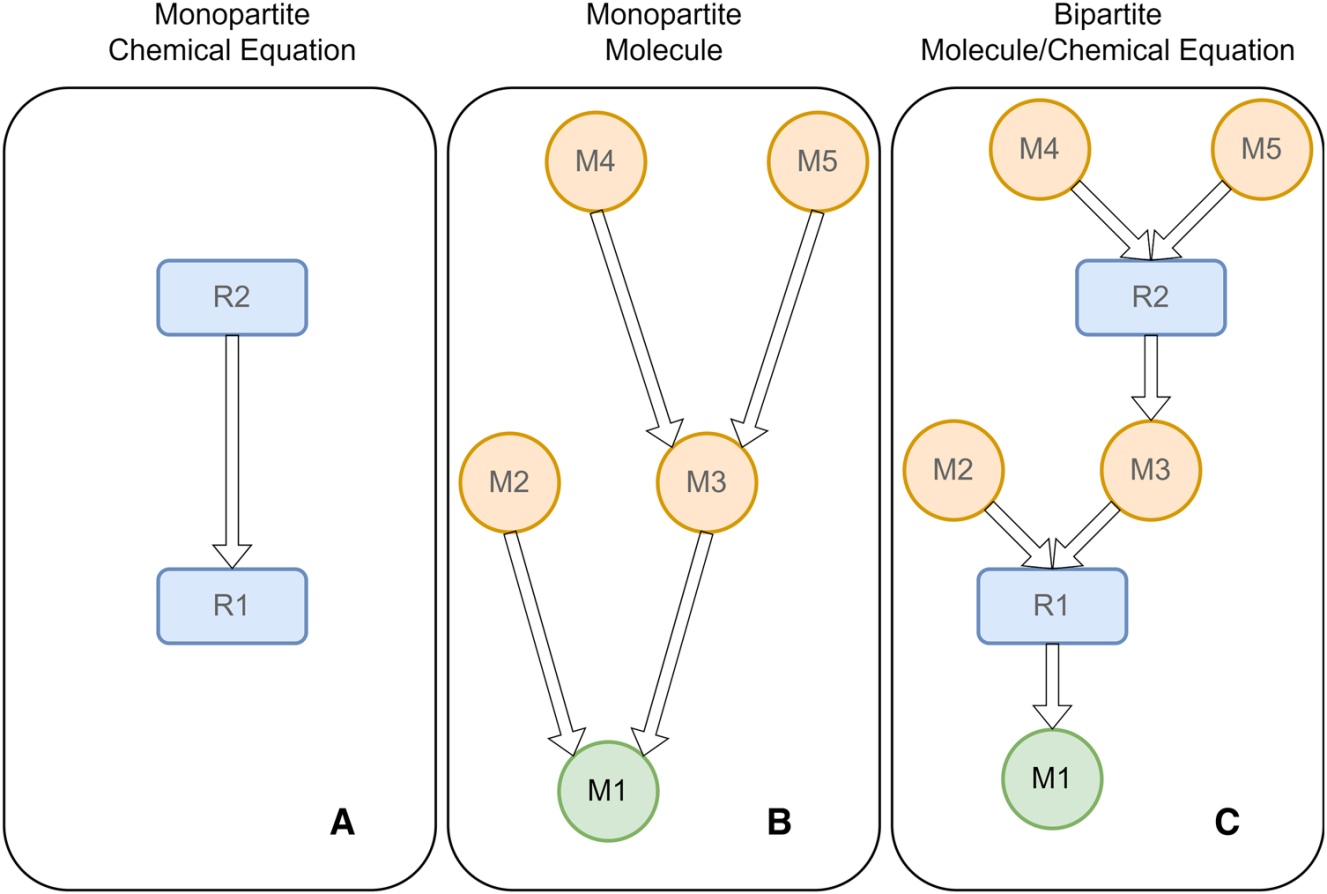
Schematic representation of different graph data models. **A** Monopartite, Chemical Equations only. **B** Monopartite, Molecules only. **C** Bipartite Molecules and Chemical Equations

SynGraph represents the connectivity between Chemical Equation and Molecule instances as a graph-like object. In a typical embodiment, based on a monopartite graph, directed edges connect Chemical Equation nodes, implying the reaction intermediates (Fig. 3 A). Alternatively, directed edges of a monopartite graph connect Molecule nodes, implicitly suggesting chemical reactions (Fig. 3 B). As a third alternative, Chemical Equations and Molecules are nodes of a bipartite graph where directed edges map role relationships (reactant, product, etc.) (Fig. 3 C). While containing the same information, these and other graph data models might serve as alternative data representations fitting specific applications.

We represent the domain logic through the following formal operations, acting on the SynGraph data model.

*Translation* Transforming SynGraph into other kind of graph objects is a crucial prerequisite for other operations. This provides an alternative to adapting the (potentially complex) application (algorithm, database, etc.) to accept the SynGraph data format and, thus, lowers the barrier to integrating third-party software.

*Identification* SynGraphs are *value objects*, and their immutable properties entirely define their nature. In the most general case, this includes the overall graph’s topology and the nodes’ properties. This means that multiple experimental synthetic routes map onto the same SynGraph (specifically, a SynRoute) if they share the same arrangement of identical reaction steps (Chemical Equations and Molecules). SynRoutes constitute complex labels for entities such as real-life synthetic routes.

*Comparison* SynGraph natively supports structural equality since it is a *value object*: to tell apart two SynGraphs is necessary and sufficient to compare the graph topology and the node properties (see above). Besides equality, SynGraph supports the calculation of structural similarity by projecting node properties across a topological distance between graphs. The following sections provide implementation specifics from a chemoinformatics standpoint.

*Merging* Merging SynGraphs corresponds to a union operation between the underlying graphs by an equality operation on the nodes. This operation is essential when creating SynTrees by merging SynRoutes, and corresponds to creating a route catalog from different CASP tools.

*Extraction* Extraction procedures yield a new SynGraph by selecting a set of nodes and edges from an existing SynGraph. Among the extraction procedures that have a scientific value, identifying distinct SynRoutes from a SynGraph is possibly the most important because it is the basis of identifying chemical routes from a route catalog (a SynTree) or the NOC.

## Implementation

### Development and deployment

The implementation information reported here provides a rationale for our software architecture choices. We refer the interested reader, the user, and the developer to the documentation enclosed with the software, where they can find more details and material aligned with code releases.

LinChemIn is a software package written in Python 3. The source code is available to the scientific community at https://github.com/syngenta/linchemin and is open to external contributions through standard git workflows. LinChemIn depends on a handful of common and reliable third-party python modules. For instance, RDKit [42] is the chemoinformatics workhorse of this application, while graph arithmetic leans mostly on NetworkX [43]. We actively manage the package dependency map, aiming at a low coupling with applications or modules that are not compatible cross-platform (e.g., GraphTools), that bring potentially conflicting second-tier library dependencies (e.g., RXNmapper [44]) or that require licenses incompatible with the MIT one. However, whenever it is not possible to avoid such applications, we prefer to wrap them into containers and expose their functionalities via dedicated REST APIs (Application Program Interfaces), letting LinChemIn satisfy their dependencies via an SDK (https://github.com/syngenta/linchemin_services).

The Object-Oriented Design (OOD) principles inspire the LinChemIn software architecture. In particular, modules and functions follow the “single-responsibility” principle, thus maximizing code reusability, facilitating testing, and supporting code refactoring. The usage of appropriate architectural patterns (e.g., the factory method pattern) ensures that the code is open for extension but closed for modification (“open-closed” principle). This approach aims to lower the barrier of incorporating external contributions to the code, thus fostering open and collaborative software development.

LinChemIn is a python library. As such, it provides front-facing interfaces to the developers of third-party applications. Several internal APIs (facades pattern) mask the complexity of the underlying code, improving its readability, reducing the coupling between the main code and applications dependent on it and decreasing the risk of breaking functionalities as the software evolves. The downside of this approach is that the high-level functions are quite large and require many parameters. To mitigate this potential burden, a set of general-purpose default values that should be suitable for many different use-cases was encoded. Moreover, each API exposes a useful helper functionality, that lists options and default for each argument, and usage examples are reported in the software documentation, helping users to navigate these large functions. In addition, LinChemIn provides a Command Line Interface (CLI) exposing high-level functionalities (e.g., route format conversion) to end-users with no software development background.

### SynGraph

**Fig. 4:**
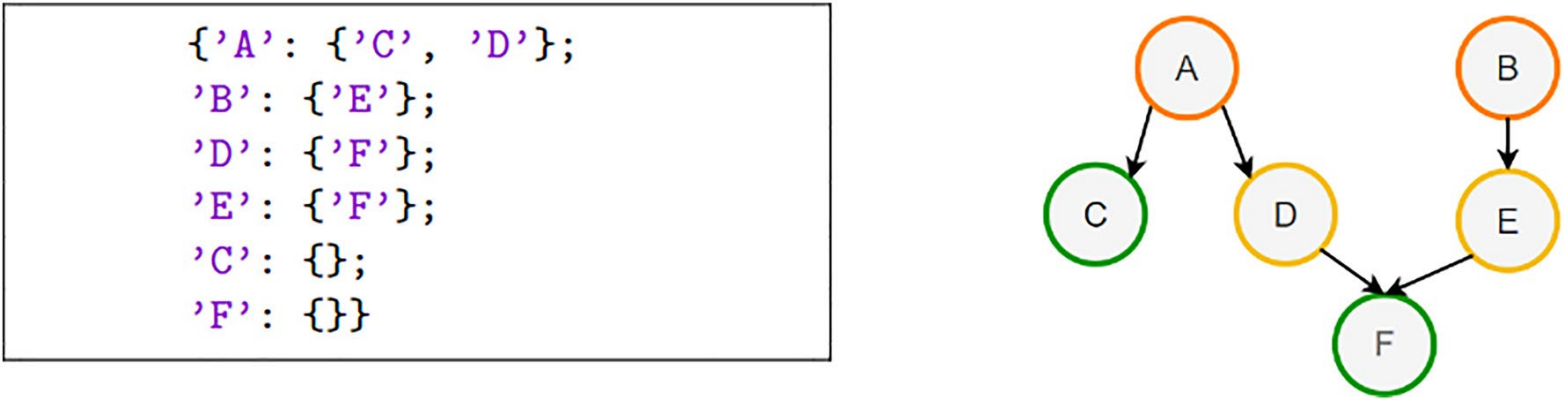
Example of SynGraph instance. *SynGraph* instance and the graphical representation of the corresponding graph.

The *SynGraph* python class, implementation of the homonym data model, is the backbone of the overall package, used as the underlying data structure for most of the code functions. It contains a graph-like structure implemented as a dictionary of sets: the key encodes a “parent” node having out edge(s), and the value is a python set containing all its “children” nodes. Using a set ensures no duplicates among the “children” nodes. While nodes are explicit, the edges stay implicit, and their direction is presumed to always be from the “parent” node to the “children” nodes (Fig. 4). This data model captures only the first mandatory ontological layer of the NOC, linking chemical reactions together via reactants and products. We leave the business logic of constructing other (optional) layers of the NOC (including reagents, procedures, etc.) to dedicated translation procedures. This approach leaves the code open to the extension to any other data models that include other objects and relationships. It reduces the need to modify the *SynGraph* class, thus risking breaking any of its many dependencies.

Instances of the *Molecule* class hold information about chemical species involved in the route. The instantiation process starts from a molecular input string (SMILES, MOL, etc.) and creates a canonicalized RDKit *Mol* instance as class attribute. This attribute is easily accessible for the dynamic calculation of molecular properties such as descriptors and fingerprints. A structure-derived molecular identifier (e.g., SMILES or InChikey string) calculated from the *Mol* attribute makes a Molecule into a *value object*, thus enabling equality assessment.

Instances of the *ChemicalEquation* class hold information about chemical reactions. The instantiation process starts from an input string (SMILES, RDMOL, etc.) and proceeds with a role attribution process and canonicalization. Ultimately, each instance contains a set of unique *Molecule* instances and a map containing information about each chemical’s roles in the reaction (reactant, reagent, product, etc.). Although the *ChemicalEquation* features other optional attributes (e.g., stoichiometry), the attribute that turns it into a *value object* is a hash of the *Molecule* instances having the role of reactants and products.

The choice of the identity attributes of *Molecule* and *ChemicalEquation* univocally echoes the identity attribute of *SynGraph* and defines the equality operations between its instances. For example, the same *SynGraph* maps two distinct routes having identical reactants and products for each reaction step, irrespective of the reagents, physical conditions, operative procedures, etc. This data model layer maps the business logic’s first layer and is open to extensions to deeper layers by including other properties of the *Molecule* or *ChemicalEquation*. Additional types of nodes and relationships can extend the data model to enable different equivalence assessments between *SynGraph* instances that include other aspects of the chemical reaction.

### Transformations

We map the synthetic chemistry domain into a graph-like data model to leverage algorithms initially developed and optimized in other fields, like social networks and sentiment analysis. Instead of adapting these algorithms to our particular scientific area’s needs (and language), LinChemIn contains flexible machinery to morph the *SynGraph* model to suit the technical requirements of specific graph libraries. This approach allows the LinChemIn user and developer to focus on the chemistry domain rather than modifying or reimplementing graph algorithms.

**Fig. 5:**
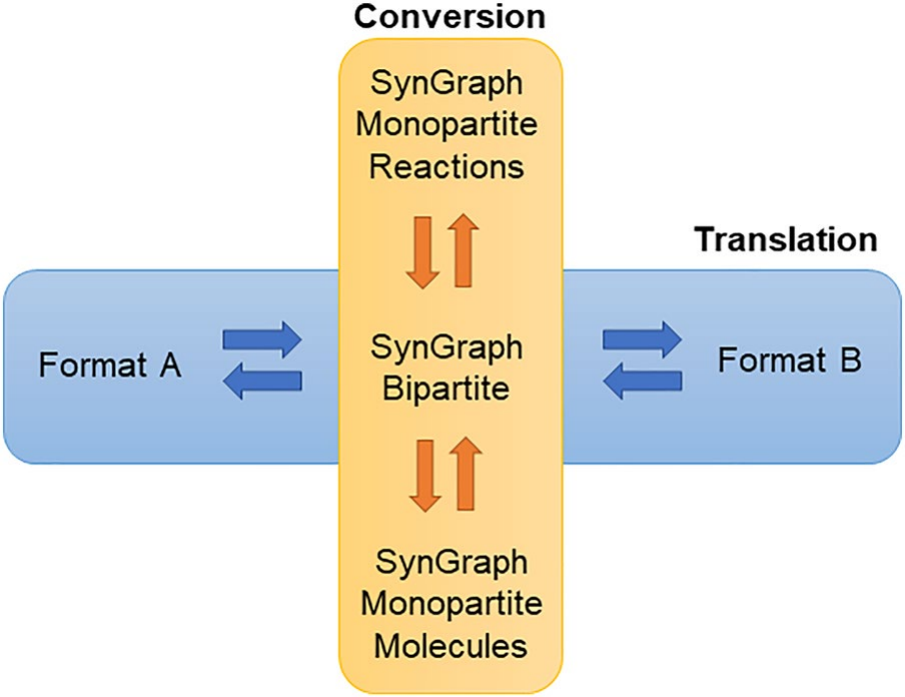
Translation and Conversion of routes. Schematic representation of the architecture relation between the LinChemIn’s modules responsible respectively for translation between format and conversion between data models

A key objective of the LinChemIn project is to read and compare synthetic routes predicted from several different CASP tools or extracted from NOC databases. Although these data are all similarly structured, with a graph-based representation, the content, the format detail, and the data model differ from case to case. The first challenge to be overcome has been to build a coherent framework in which routes generated by different sources are homogeneous and can thus be compared. A machinery to transform routes was needed. However, the route transformation has actually two components: on the one hand, it must be possible to “translate” graph objects between data formats (e.g., from the output format of a CASP tool to a Networkx object) and, on the other hand, the possibility to “convert” graph objects between data models (e.g., from a monopartite - molecules only representation to a bipartite - molecules and reactions one) is also needed. In LinChemIn, these two types of transformation are treated independently, so that the combinatorial explosion of the number of possible combinations deriving from mixing and matching data formats and data models is avoided, while keeping full flexibility in selecting the most suitable combination. The overall transformation proceeds through a sequence of changes of format, from the selected input to the selected output, that always includes SynGraph. The latter is the only format that handles the conversion between data models, while also guaranteeing the standardization of chemical information. A schematic representation of the orthogonal handling of translation and conversion is shown in Fig. 5. The architecture of the module allows the developer to easily expand the list of supported data formats, that already includes popular third parties formats, such as Networkx and Pydot, with additional formats and easily project the existing data models onto the new formats. Conversely, the user can create bespoke data transformation workflows by combining available data formats and models to suit specific applicative or visualization needs.

### Operations on routes

Besides data I/O and data transformation, LinChemIn enables operations on connected synthetic datasets. For instance, the design of *SynGraph* natively supports smart data set merging, leveraging the node equality properties to identify duplicates and super-/sub-sets. This is important while integrating the output of different CASP tools to yield synthetic trees that can include human input and experimental data extracted from NOC. The identification of the intersection between *SynGraph* instances is enabled by the equality properties of the nodes (*Molecule* and *ChemicalEquation*) underlying the graph representation of the synthetic routes. It is possible to configure these properties to obtain different behavior upon merging. For instance, the tautomeric differences between molecules are ignored by switching the equality property from a canonical isomeric SMILES string to an InChi key string. Alternatively, removing the isomeric information leads to different streoisomerically related compounds collapsing as the same node. This functionality relies on a set of molecular hashes available in RDKit, and the developer can extend this set to suit specific needs.

In addition, LinChemIn allows the calculation of route-level descriptors and the comparison between routes. In the next section, we discuss both cases with illustrative examples. The first release of the code features a small number of route descriptors, and while we are developing more complex ones, we look forward to the user community feedback and contribution.

### Route descriptors

We extend the definition of descriptors from the molecule [45] to the synthetic route. Thus, the route descriptor is “the final result of a logical and mathematical procedure, which transforms chemical information encoded within a symbolic representation of a synthetic route into a useful number or the result of a standardized experiment.” This definition encompasses computed, modeled, and experimental descriptors. An example is the LogP, a quantitative representation of the lipophilicity of the molecules that can be measured in an experiment or predicted with a model. Similarly, the overall chemical yield can be the outcome of an experimental synthetic procedure or a route projection of the yield predicted for the single steps [46–50]. Here, we describe our approach to calculate route descriptors. In a future code release, we will include methods to combine descriptors and construct hybrid experimental/modeled route metrics.

**Fig. 6:**
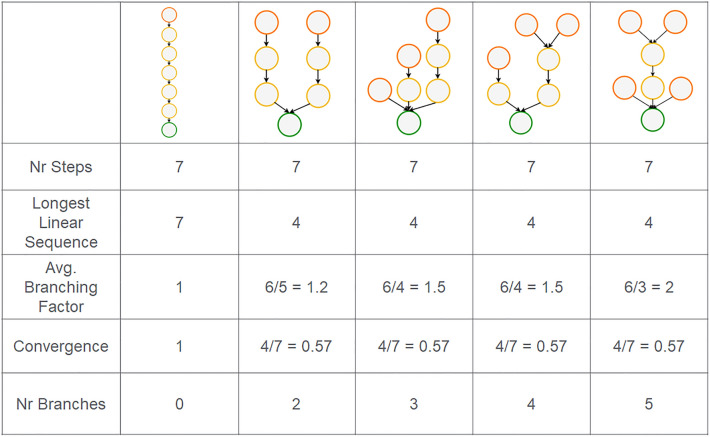
Route descriptors. The value of structural descriptors calculated on representative graphs illustrates their discriminative capability

LinChemIn features an extensible factory of methods that take as an input the *SynGraph* and return numerical or Boolean values of route descriptors. Each descriptor captures a particular aspect of the route composition and structure, leveraging the graph-like architecture underlying the *SynGraph* instances used to represent routes. In Table 1, we list the main descriptors currently implemented in the LinChemIn library, while Fig. 6 highlights how each descriptor is able to discriminate between particular graph structures.

**Table 1:** Descriptors currently implemented in LinChemIn and how they are measured

Descriptor	What is measured	How it is measured
Number of Steps	Overall size of the route	Number of unique ChemicalEquation nodes in a monopartite reaction-only SynRoute
Longest Linear Sequence	Length of the longest synthetic branch connecting the root with a starting material.	Number of unique ChemicalEquation nodes included in a monopartite reaction-only SynPath
Number of Branches	Number of times a synthetic route departs from linearity	Number of ChemicalEquation nodes whose ChemicalEquation “child” node is also a “child” of other ChemicalEquation nodes.
Convergence [51]	Degree of branching of a route	Ratio between the longest linear sequence and the number of steps in a monopartite reaction-only SynRoute.
Average Branching Factor	Degree of branching of a route	Ratio between the number of non-root nodes and the number of non-leaf nodes in a monopartite reaction-only SynRoute.

### Comparison between routes

*Equivalence* From a chemical standpoint, we call “identical” two synthetic routes that share the same synthetic steps arranged in the same order. However, this simple definition depends on the level of detail adopted to describe each step. For example, we can tell apart two identical reactions (same reactants/products) if we consider reagents, procedures, or operational aspects (user, date, chemical batches, etc.); this difference also echoes at the route level. For this reason, we prefer the term route equivalence to that of identity and link it to the level of detail each route holds. As described above, we arbitrarily align the *SynGraph* equality property to the route equivalence level that captures the single reactions’ reactants and products. This decision aligns with the formal treatment of retrosynthesis provided by Corey [52] and Hoffman [53] and ensures the best compatibility across CASP tools and NOC databases. The *SynGraph* (a *value object*) is thus a label for synthetic routes (*entities*). The other layers of route equivalence find their place in the data model but do not contribute to the route “label.” We build the route identity logic on the equality of the dictionaries of sets that constitute *SynGraph*. Two dictionaries are identical when they have the same key-value pairs. Since both keys and values are *Molecule* or *ChemicalEquation* instances (*value objects*), the *SynGraph* equivalence conveniently relies on standard python compassion functions.

**Fig. 7:**
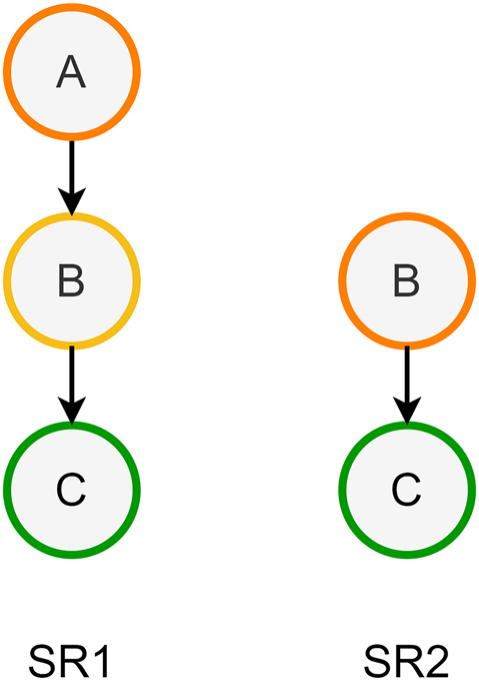
Sub- and Super-set. Depiction of the subset concept: the SR2 on the right is a subset of SR1 on the left

*Sub-/Super-Set* We consider a synthetic route (SR2 in Fig. 7) to be a subset of another synthetic route (SR1) if it shares only a portion of the synthetic steps. The case where the target compound is shared is chemically relevant, and it represents the case when upon truncating a route branch, an intermediate is sourced rather than synthesized. The sub-set assessment between routes efficiently leverages the underlying dictionary data structure of *SynGraph* rather than relying on potentially expensive graph isomorphism algorithms.

*Similarity* The equivalence (and the identity) is a Boolean response obtained by an equality function. On the contrary, the similarity is a numerical response obtained by comparing the numerical properties of two objects. By extending the molecular similarity approach to routes, we could compare the numerical values of route descriptors or hash the descriptors into route fingerprints and compare these instead. Unfortunately, the number of route descriptors is still limited, and their importance is still unclear. For this reason, we privilege a definition of similarity that depends explicitly on the structure of the route and the chemical nature of its steps.

LinChemIn leverages the Graph Edit Distance (GED) to compute the similarity between synthetic routes. The GED between two graphs, G1 and G2, is the cost of the optimal edit path needed to transform G1 into a graph isomorphic to G2. The edit path may involve three types of operations, node insertion, node deletion, and node substitution, each associated with a specific ’cost.’ The GED is a graph arithmetic operation analogous to the Levenshtein distance between strings [43], and it does not take into direct consideration the chemical information, which however is essential when comparing synthetic routes. Genheden et al. [54] introduced chemical information in the GED by enabling the GED cost functions to include calculations of chemical similarity at molecular and reaction levels.

Inspired by this approach, we implemented a simple framework of route similarity relying on the NetworkX GED algorithm. The NetworkX GED functionality accepts a bespoke cost function for the node substitution through the parameter ’node_subst_cost’ of the ’graph_edit_distance’ or ’optimized_graph_edit_distance’ functions. Here, the cost for deleting and inserting nodes was set to unity, as this is common in literature and corresponds to swapping a node with one of a different type (e.g., Molecule with a Chemical Equation). Otherwise, the cost for node substitution derives from fingerprint-based molecular or reaction similarity, as implemented in RDKit. Since the LinChemIn module exposes the RDKit options, the user can select the most appropriate combination of reaction/molecular fingerprint and similarity metric. By restricting the application to rooted graphs (such as synthetic routes), the ’roots’ parameter of the GED function increases the computational efficiency of the algorithms.

**Fig. 8:**
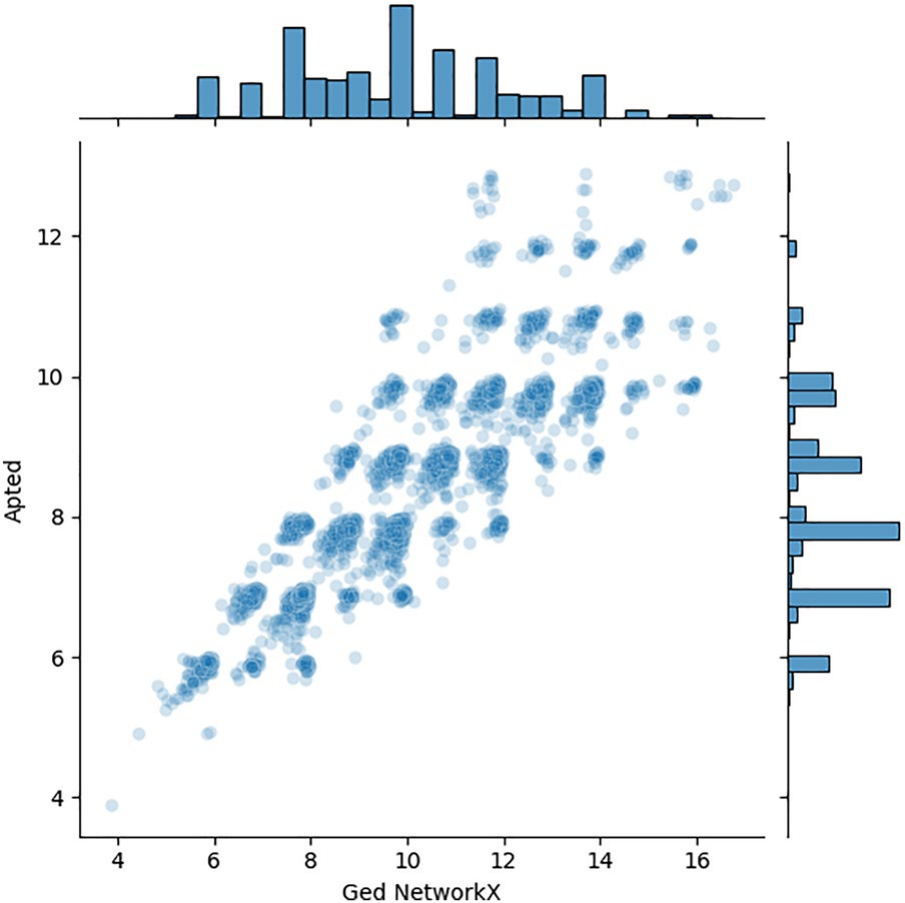
GED vs APTED correlation. Relation between the GED values computed with the NetworkX algorithm and with the APTED algorithm. The Spearman correlation between the two sets is 0.87

**Fig. 9:**
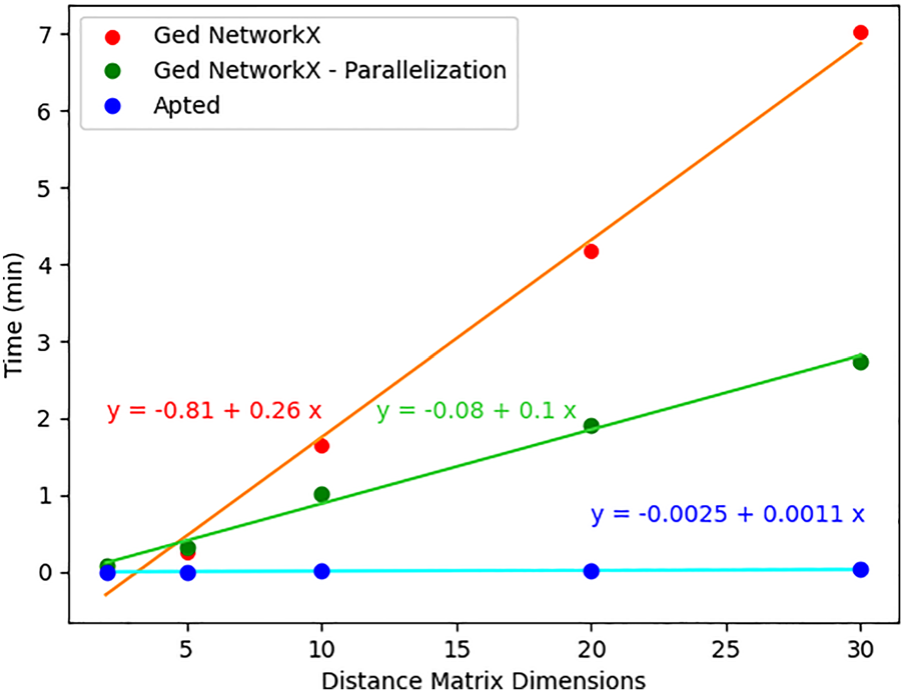
GED vs APTED computational efficiency. Computational time needed to compute the distance matrix for an increasing number of routes with APTED and NetworkX algorithms

To our knowledge, the only currently available open-source package to compute the similarity between Synthetic Routes is the one developed by Genheden et al. [54] using the APTED algorithm [55, 56]. We provide a comparison between that approach and the one described here. However, it is worth noticing that the absence of a reference ground truth for synthetic routes similarity does not allow one to determine whether one implementation is more accurate than the other. We took 100 routes among those used by Genheden et al. [57, 58] and we computed the distance matrix with both the APTED and the NetworkX algorithms using the monopartite representation (chemical reaction only) of the routes. To make the results comparable, we used a set of parameters for the chemical similarity calculations analogous to the one used by Genheden et al.: difference fingerprints for chemical reactions, Morgan fingerprints for the molecules, and the Tanimoto algorithm for the similarity. Figure 8 shows a good correlation between the values computed with the two algorithms, although the APTED values are generally lower than those calculated with NetworkX, and the agreement decreases as the values increase, suggesting a variance accumulation. On the one hand, the difference in the absolute values could depend on the actual algorithm. Also, in the approach by Genheden et al., some heuristics were imposed over the APTED algorithm to compensate for the fact that the routes are not ordered trees. On the other hand, small differences in the RDKit parameters used lead to different values of chemical similarity and, in turn, to different values of GED. Even though the absolute values are not identical, the overall Spearman correlation between the two sets of APTED and GED values is good, namely 0.87. For what concerns computational efficiency, we compared the time required by the two algorithms to compute the distance matrix for an increasing number of routes. The distance matrix is an $n \times n$ symmetric matrix, where *n* is the number of routes in the considered set. As shown in Fig. 9, the algorithm by Genheden et al. outperforms the NetworkX GED: although both algorithms have an approximately linear dependency on the distance matrix dimensions, the slope for the APTED approach is about two orders of magnitude smaller than the one for the GED NetworkX. The performance of the NetworkX algorithm can be improved by using the parallelization option for calculating the distance matrix available in LinChemIn.

*Clustering* LinChemIn allows to cluster synthetic routes based on their similarity matrix. Currently, two clustering algorithms are available: Agglomerative Clustering [54] as implemented in sklearn, and Hdbscan [59]. Since the agglomerative clustering algorithm needs the number of clusters as input, we followed the strategy of Genheden et al. [54] and implemented a process to optimize this parameter based on the Silhouette score. The default linkage is single, but users can modify it according to their needs. The ’clustering’ module has a factory structure to enable scientists to experiment with other clustering algorithms.

## Results and discussion

In this section, we present some code snippets providing practical applications of LinChemIn and an example of how a user could implement the calculation of a custom metric. A high-level facade function wraps the main functionalities and simplifies their usage; a convenient helper supports interactive usage (e.g., via Jupyter) by exposing detailed information about available methods, parameters and their default values. Moreover, an applicative case study is provided as Additional file 1.

### Translation and conversion functions

Using LinChemIn, the users can handle the outputs of various CASP tools and transform the format and the data model of the predicted routes. The following code shows how to exploit the main facade function ’processs_routes’ to handle the predictions of two different CASP tools, AiZynthFinder [17] and IBMRXN [18], provided as JSON files, translate them into a list of *SynGraph* instances, while also changing the type of graph; the routes are then written into a json file as lists of dictionaries containing nodes and edges.

The input files are passed to the function alongside the names of the CASP tools that generated them through a dictionary of the form {’file_path’: ’casp_name’} By default, a json file containing the bipartite representation of the routes is created. However, it is possible to change the output file format to csv through the ’output_format’ argument and to select a different data model by specifying the ’out_data_model’ parameter. The ’process_routes’ function also creates an output object, whose attributes store the outcomes of the calculation as object.

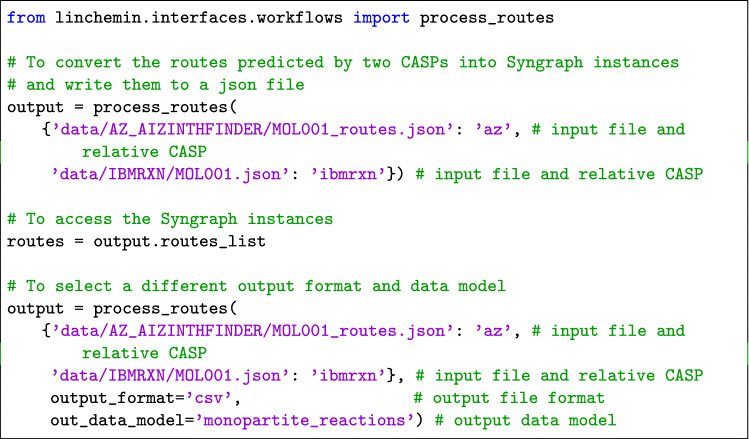



### Routes operations

The ’process_routes’ function allows users to select one ore more operations to be performed on the routes, such as the calculating descriptors or clustering, by specifying the ’functionalities’ argument as a list of strings.

*Routes Descriptors* The code below shows how to compute some descriptors of routes generated by different CASP tools. This is done by specifying the ’compute_descriptors’ string in the ’functionalities’ list. By default all the available descriptors are calculated, however it is possible to specify the desired ones by passing their names to the ’descriptors’ parameter as a list of strings. The output is stored in the ’descriptors’ attribute of the output object as a pandas Dataframe, and it is also written to the ’descriptors.csv’ file. Moreover, it is possible to use parallel computing by setting the argument ’parallelization’ to True and selecting the number of CPUs to be used through the ’n_cpu’ parameter.

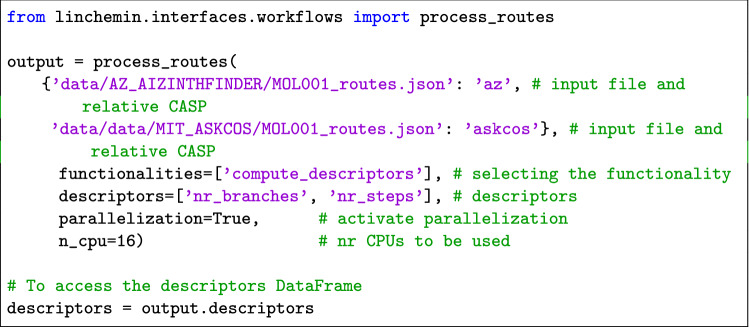



*Routes Clustering*

Another available functionality is the one for clustering the routes based on their GED. Once again, the facade function ’process_routes’ is the simplest way to access this functionality.

The input json files are passed to the function through a dictionary, as previsouly shown, while the functionality to be specified is ’clustering’. The clustering algorithm can be selected through the ’clustering_method’ argument; by default the Agglomerative Clustering algorithm is used when there are less than 15 routes in the input list and Hdbscan otherwise. The ’ged_method’ parameter determines which algorithm is used to compute the similarity matrix; by default, the standard GED algorithm provided by NetworkX is selected. It is also possible to specify the type of fingerprints and similarity method for both molecules and chemical reactions by setting the ’ged_params’ parameter.
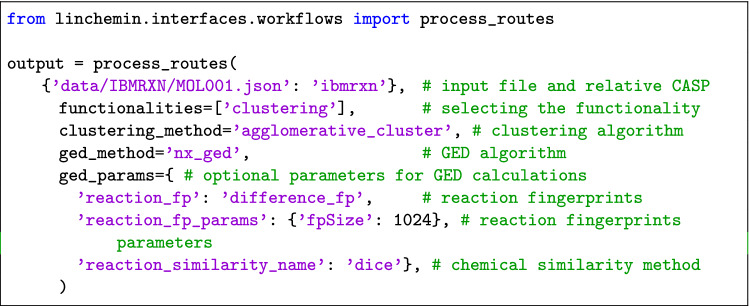


### Code customization

The module ’route_descriptors’ dedicated to the calculation of route descriptors has been implemented as a factory pattern and the functions computing the metrics are methods of subclasses of the abstract class *DescriptorCalculator*. Thus, to introduce a new metric, the first step is to define a new subclass, for example *CustomMetric(DescriptorCalculator)*, and its method ’compute_descriptor’. The latter should contain the code necessary to compute the new metric and return its value. As can be seen in the snippet below, there are no requirements for the type of input of the method and the developer can build it as they prefer.
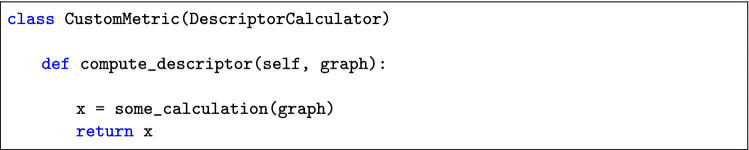


The second step is to add the new metric among the available ones. This can be done by adding a new key in the ’route_descriptors’ dictionary of the *DescriptorCalculatorFactory* class, which maps a string identifying a metric into the relative subclass; the string is then used to access the metrics through the descriptor_calculator function, as shown below.
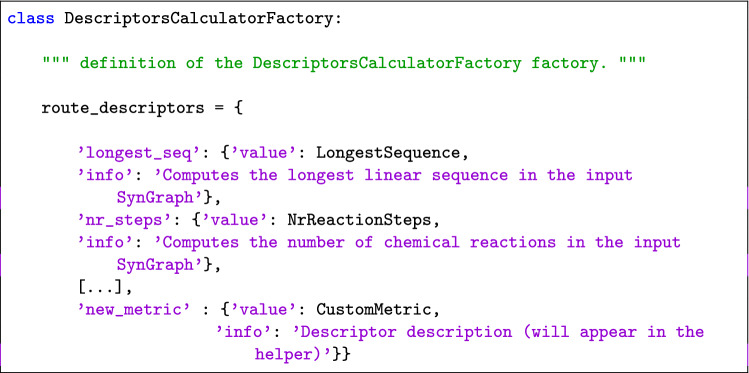


The usage will then simply be:
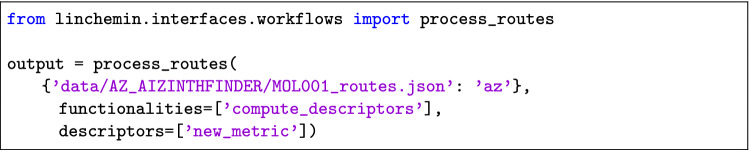


Most of the modules in the package have the same factory structure as the ’route_descriptors’, so that the procedure to add new functionalities also in other parts of the code (format conversion, ged calculation and clustering) is the same as the one shown above.

## Conclusions

We introduced LinChemIn, an open-source python toolkit to work with synthetic routes generated by a variety of different sources, such as CASP tools and NOC. The toolkit enables interconversion between data formats and data models, as well as route-level analysis and operations. The Object-Oriented Design principles inspire the software architecture so that functions and modules are structured using appropriate architectural patterns to maximize code reusability and support code testing and refactoring. This also aims to minimize the effort needed to incorporate external contributions and to encourage open and collaborative software development. Moreover, the Domain-Driven Design principles were adopted to ensure a close match between the scientific domain (i.e., synthetic chemistry) and the structure and language of the code, fostering a constructive collaboration between technical and domain experts.

We presented the data models created to map concepts relevant for the synthetic chemistry domain, as well as the main functionalities that have been implemented so far. The current version of LinChemin represents the first step of a much bigger project, aiming to build an entire “ecosystem” of data models and functionalities to manipulate and operate on synthetic routes, similar to what RDKit and other tools created for molecules.

In future releases of the code, we aim to include more sophisticated synthetic routes metrics integrating experimental and modeled data, a multi-parameter score system and a plug-in to directly connect LinChemIn to an NOC database.

## Availability and requirements

LinChemIn is available at https://github.com/syngenta/linchemin Programming language: Python >=3.9 License: MIT Other requirements:

- rdkit ≥ 2022.3
- rdchiral
- pydot
- networkx
- pandas
- numpy
- hdbscan
- scikit-learn
- joblib == 1.1.0

Scripts and data used to generate the plots are available at https://github.com/syngenta/LinChemIn_publications Services accessible via API are available at https://github.com/syngenta/linchemin_services

## Supplementary Information

**Additional file 1: Fig S1. **Molecular structure of Amenamevir. **Fig S2.** Example of identified subset. On the right, the route predicted by AiZynthFinder and on the left one of those generated by IBMRXN. The red circle highlights the portion of the IBM route identical to the route from AiZynthFinder. **Fig S3. **Distance matrix heatmap for the 64 routes in the considered set. **Fig S4.**Pair of routes that are not identical but for which the GED weighted with the chemical similarity is zero. **Fig S5.**Dendrogram for the clustering of the routes. **Fig S6.**Clustering visualization of the routes. The axes have been arbitrarily chosen as the distances of each route from the first, the 33rd and the 61st route in the distance matrix. **Fig S7.**Representative route for cluster 0. **Fig S8.**Most representative route for cluster 1. **Fig S9.**Most representative route for cluster 2. **Fig S10.**Most representative route for cluster 3. **Fig S11.**Most representative route for cluster 4. **Fig S12.**Most representative route for cluster 5. **Fig S13.**Most representative route for cluster -1 (route classified as "noise"). **Table S1.**Parameters used in computing the clustering of the routes. **Table S2.**Information about the routes' clusters. For each cluster the number of routes, the average number of steps and the average number of branches are reported.

## Footnotes

1 Here, the term Chemical Equation only refers to the structural part of a Chemical Reaction. We realized that the term Reaction, or Chemical Reaction hosts a range of different meanings, depending on the context, on the source, and the usage. Thus, we decided to reserve the term Chemical Reaction for a different ontological level, which includes other layers of information (e.g., reaction condition, experimental details, etc...)
