# Supplementary material for: LinChemIn: SynGraph—a data model and a toolkit to analyze and compare synthetic routes
Source: J Cheminform. 2023 Apr 1;15:41. doi: 10.1186/s13321-023-00714-y (PMC10067316; doi:10.1186/s13321-023-00714-y)
Supplement: Supplementary file 1 — Additional file 1: Fig S1. Molecular structure of Amenamevir. Fig S2. Example of identified subset. On the right, the route predicted by AiZynthFinder and on the left one of those generated by IBMRXN. The red circle highlights the portion of the IBM route identical to the route from AiZynthFinder. Fig S3. Distance matrix heatmap for the 64 routes in the considered set. Fig S4.Pair of routes that are not identical but for which the GED weighted with the chemical similarity is zero. Fig S5.Dendrogram for the clustering of the routes. Fig S6.Clustering visualization of the routes. The axes have been arbitrarily chosen as the distances of each route from the first, the 33rd and the 61st route in the distance matrix. Fig S7.Representative route for cluster 0. Fig S8.Most representative route for cluster 1. Fig S9.Most representative route for cluster 2. Fig S10.Most representative route for cluster 3. Fig S11.Most representative route for cluster 4. Fig S12.Most representative route for cluster 5. Fig S13.Most representative route for cluster -1 (route classified as "noise"). Table S1.Parameters used in computing the clustering of the routes. Table S2.Information about the routes' clusters. For each cluster the number of routes, the average number of steps and the average number of branches are reported. [file 13321_2023_714_MOESM1_ESM.docx]

LinChemIn:SynGraph. A data model and a toolkit to analyze and compare synthetic routes

By Marta Pasquini, Marco Stenta

# S1. Additional file

As case study to demonstrate the usage of our Python toolkit LinChemIn, we considered the anti-viral drug Amenamevir (Figure S1, [https://pubchem.ncbi.nlm.nih.gov/#query=MNHNIVNAFBSLLX-UHFFFAOYSA-N](https://pubchem.ncbi.nlm.nih.gov/%23query=MNHNIVNAFBSLLX-UHFFFAOYSA-N)) and used three different CASP tools (IBMRXN, Askcos and AiZynthFinder) to predict routes for it. All data and scripts used for this case study are available at https://github.com/syngenta/LinChemIn\_publications

A total of 64 different routes were predicted. No duplicated routes were identified, while a set of subsets were found: one of the routes predicted by AiZynthFinder is subset of various routes predicted by Askcos (Figure S2). This could be caused by a different set of starting materials used in the two CASP tools.


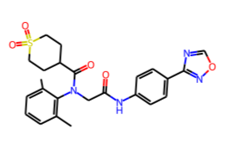


Figure S1: Molecular structure of Amenamevir.


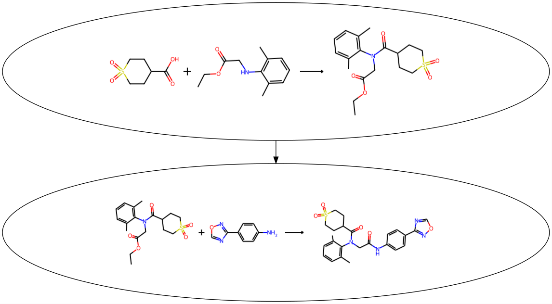

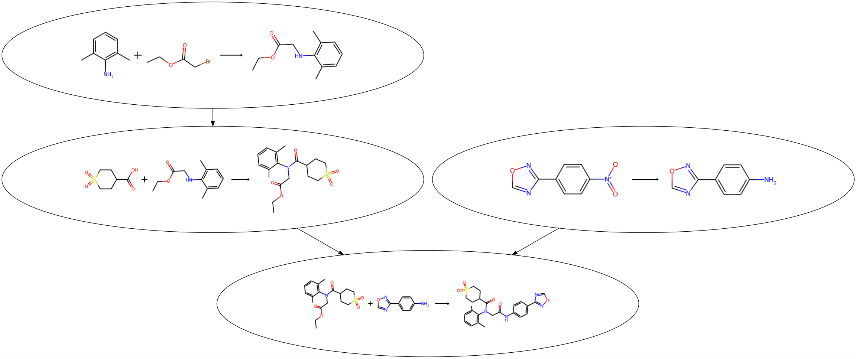


Figure S2: Example of identified subset. On the right, the route predicted by AiZynthFinder and on the left one of those generated by IBMRXN. The red circle highlights the portion of the IBM route identical to the route from AiZynthFinder.

The distance matrix for the set of routes was computed and, based on it, the routes were clustered with the Hdbscan algorithm. In Table S1 the parameters used in the calculations are reported. Figure S3 shows the distance matrix relative to the set of routes as a heatmap. Few distance values equal to zero appear also off-diagonal, indicating that there are pairs of routes for which the Graph Edit Distance weighted by the chemical similarity is zero, even if they are not technically identical (indeed, no duplicates were found). These are pairs of routes that only differ for how a certain regent is specified (e.g., HCl vs Cl^-^); an example of such routes is reported in Figure S4.

The algorithm identified 6 different clusters and grouped some routes as “noise” (cluster -1). Table S2 reports the number of routes, the average number of steps and the average number of branches for each cluster. Figure S5 shows the dendrogram of the final clustering, while in Figure S6 the graphical representation of the clustering is shown.

For each cluster, we also identified a “representative” route as the route closer to the center of the cluster; pictures of these routes are shown in Figures S7-13.

Table S1: Parameters used in computing the clustering of the routes.

| **Parameter** | **Value** |
| --- | --- |
| Clustering algorithm | hdbscan |
| Minimum cluster size | 5 |
| GED algorithm | Networkx’s GED |
| Reaction Fingerprint type | structural |
| Reaction Fingerprint parameters | None (default have been used) ^a^ |
| Reaction similarity method | tanimoto |

^a^ RDKit default values as described here: https://github.com/rdkit/rdkit/blob/master/Code/GraphMol/ChemReactions/ReactionFingerprints.cpp#L123


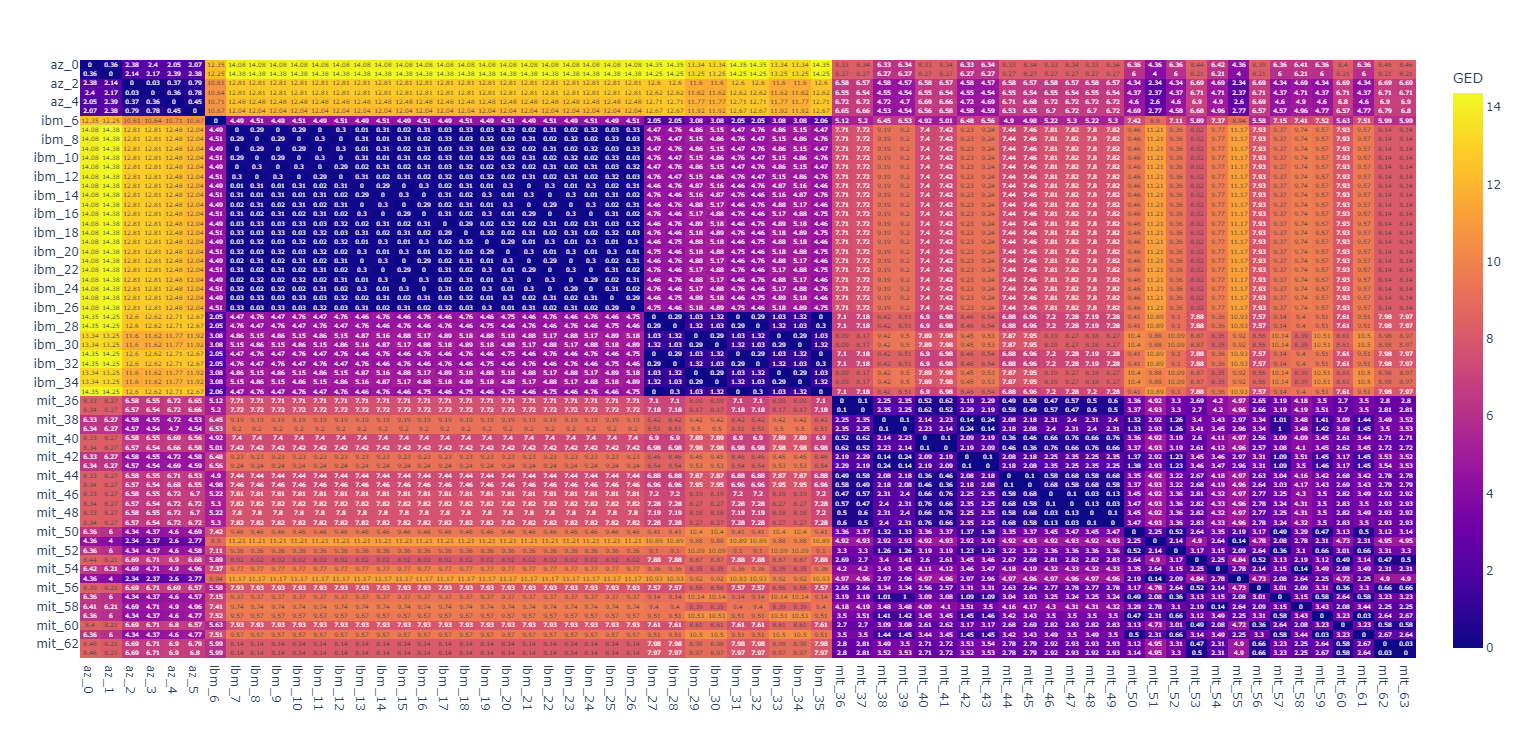


Figure S3 Distance matrix heatmap for the 64 routes in the considered set.


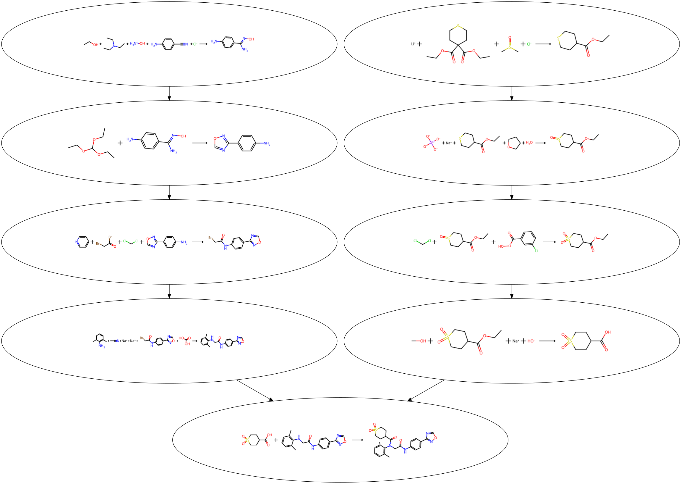

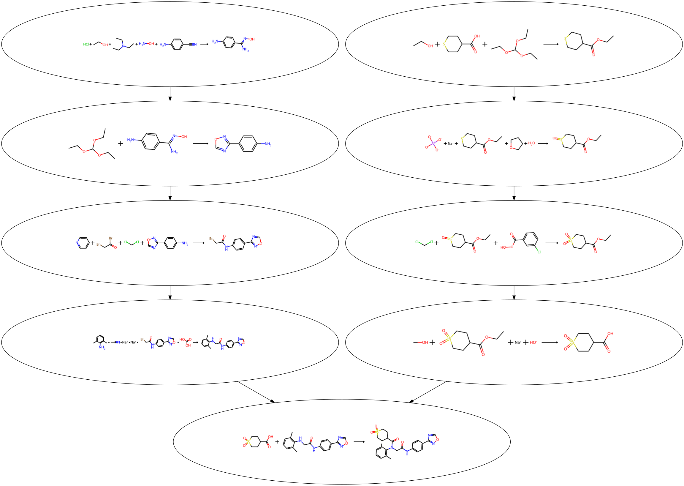


Figure S4: Pair of routes that are not identical but for which the GED weighted with the chemical similarity is zero.

Table S2: Information about the routes' clusters. For each cluster the number of routes, the average number of steps and the average number of branches are reported.

| **Cluster Label** | **Nr Routes** | **Avg Nr Steps** | **Avg Nr Branches** |
| --- | --- | --- | --- |
| -1 | 8 | 3.00 | 0.5 |
| 0 | 7 | 5.71 | 2.0 |
| 1 | 10 | 8.50 | 3.8 |
| 2 | 10 | 9.00 | 2.0 |
| 3 | 10 | 9.00 | 2.0 |
| 4 | 9 | 5.00 | 2.0 |
| 5 | 10 | 6.00 | 2.0 |


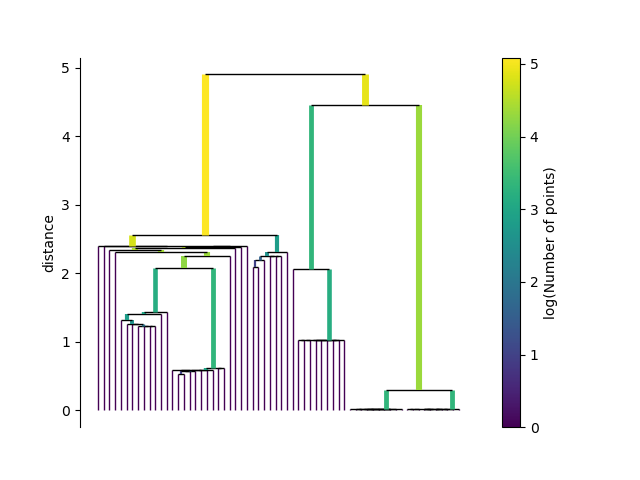


Figure S5 Dendrogram for the clustering of the routes.


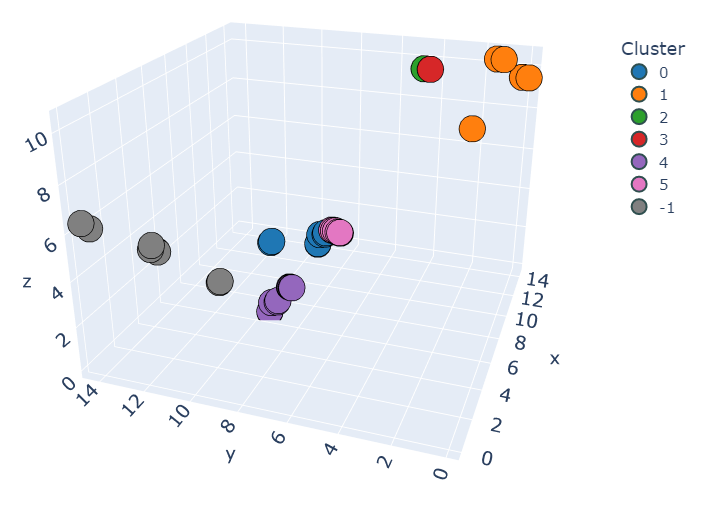


Figure S6: Clustering visualization of the routes. The axes have been arbitrarily chosen as the distances of each route from the first, the 33^rd^ and the 61^st^ route in the distance matrix.


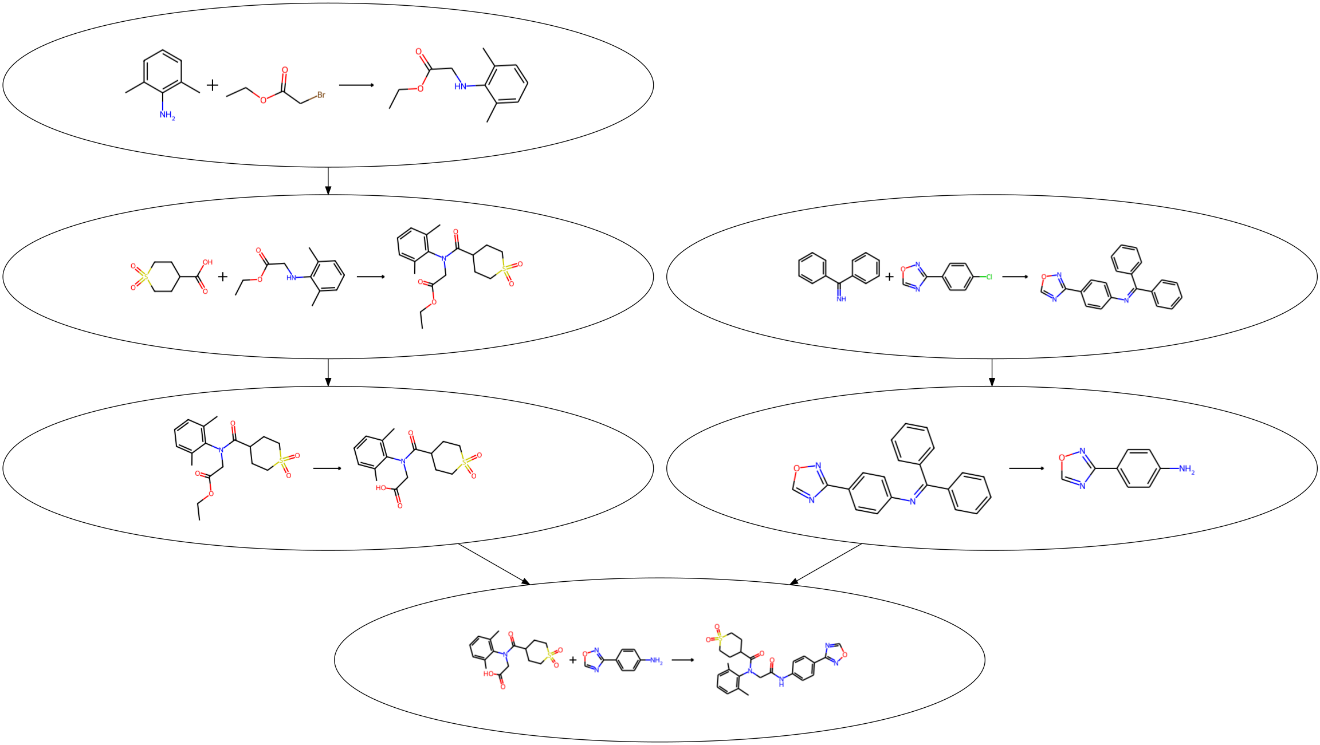


Figure S7: Representative route for cluster 0.


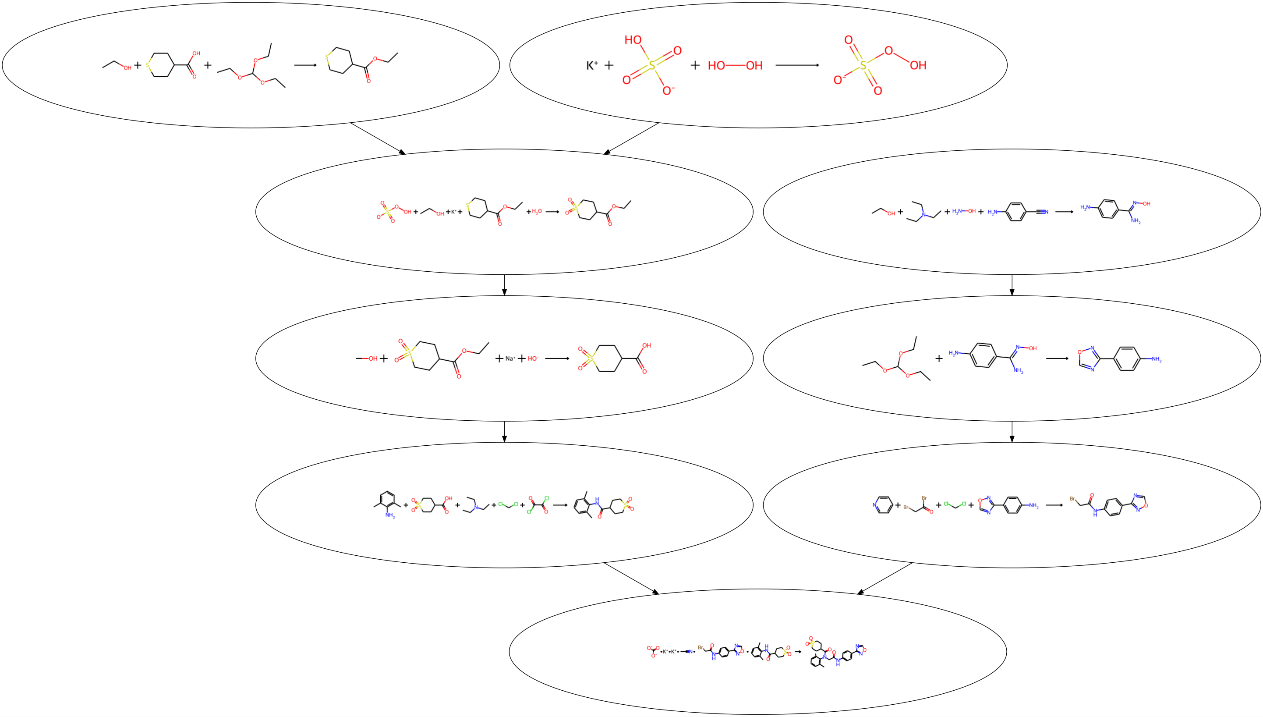


Figure S8: Most representative route for cluster 1.


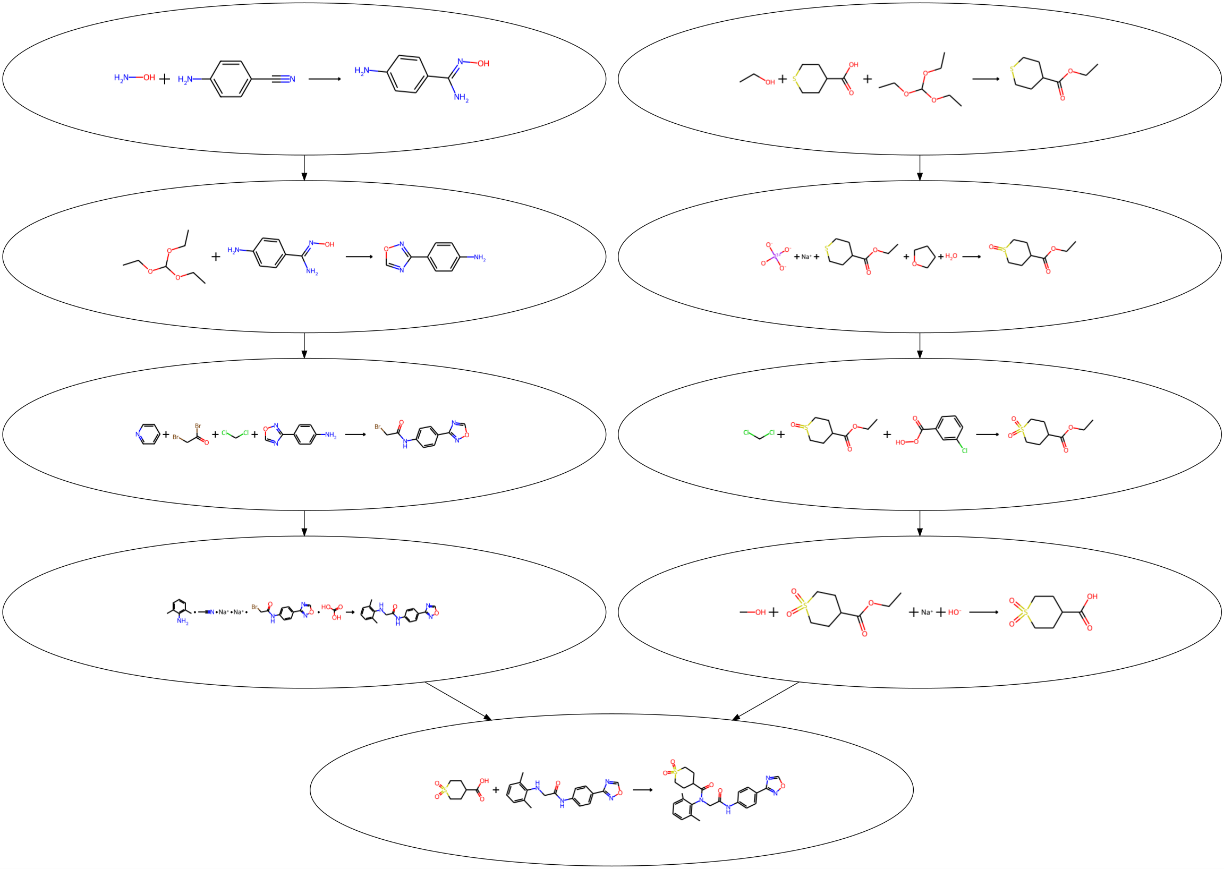


Figure S9: Most representative route for cluster 2.


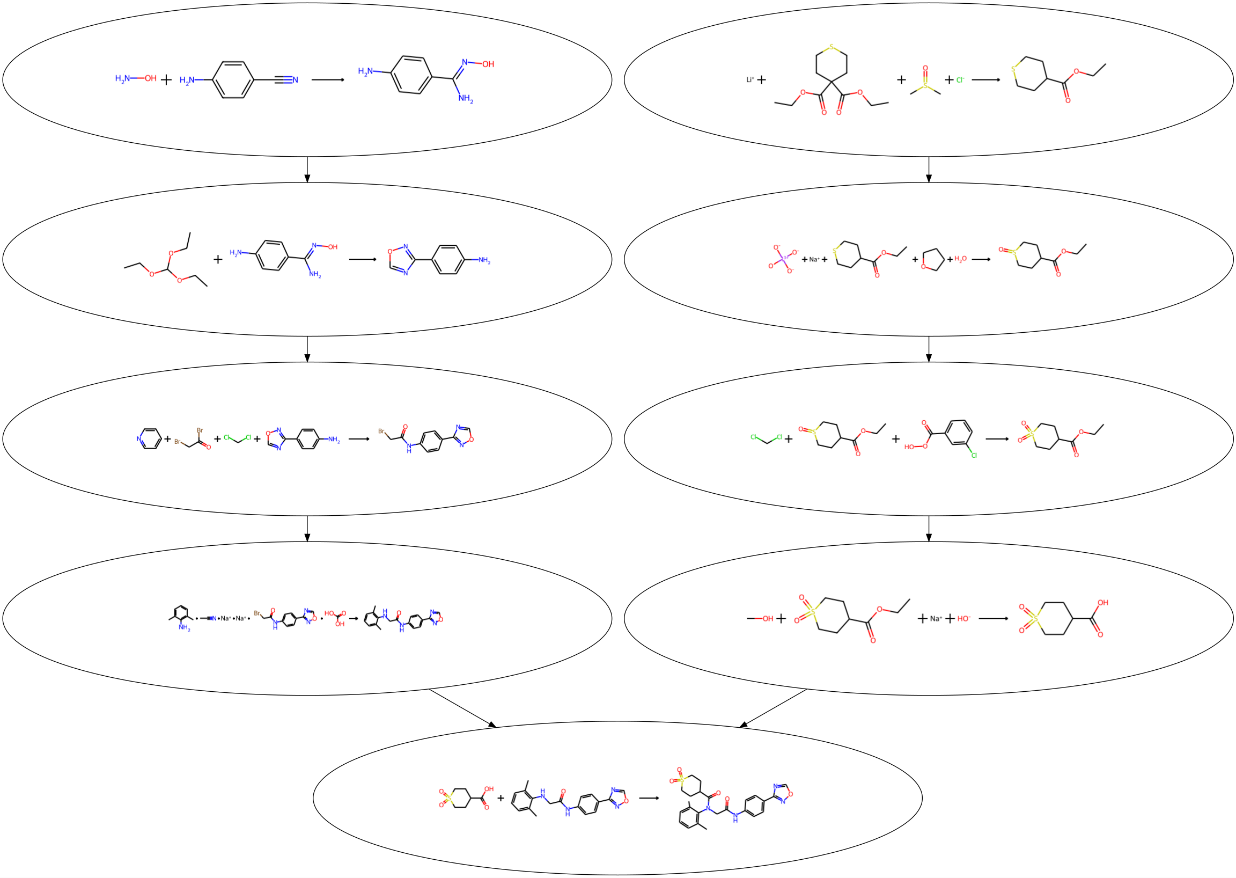


Figure10: Most representative route for cluster 3.


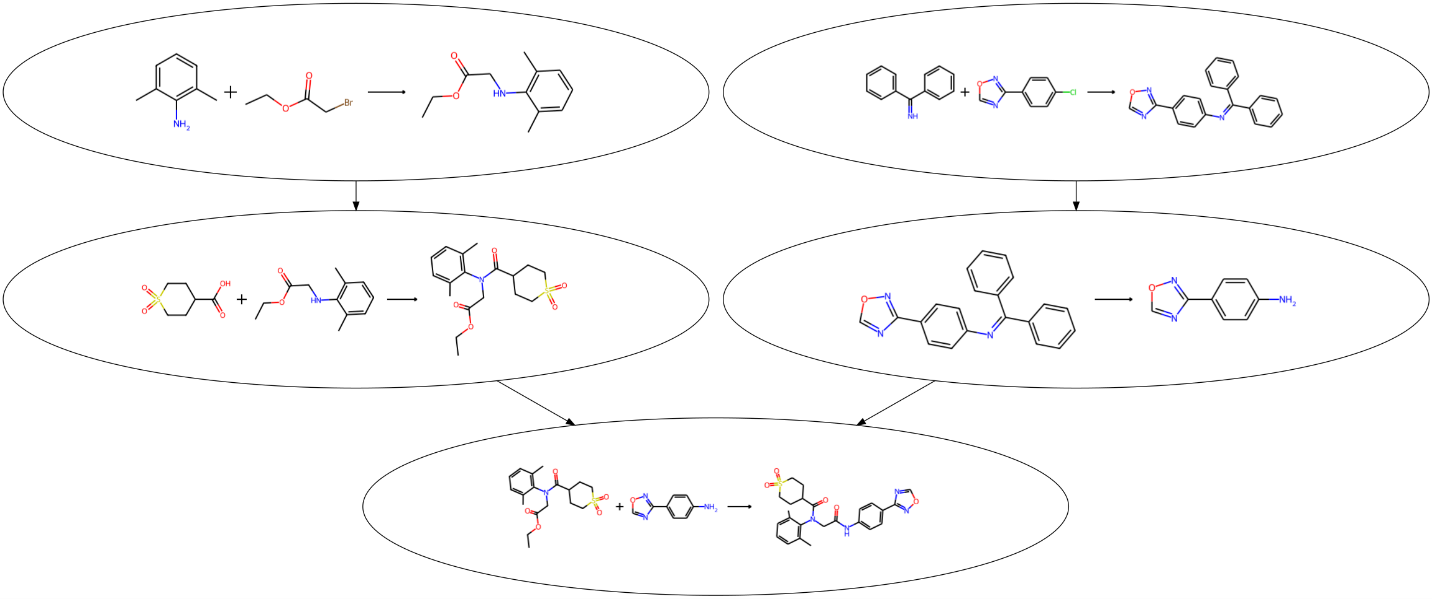


Figure S11: Most representative route for cluster 4.


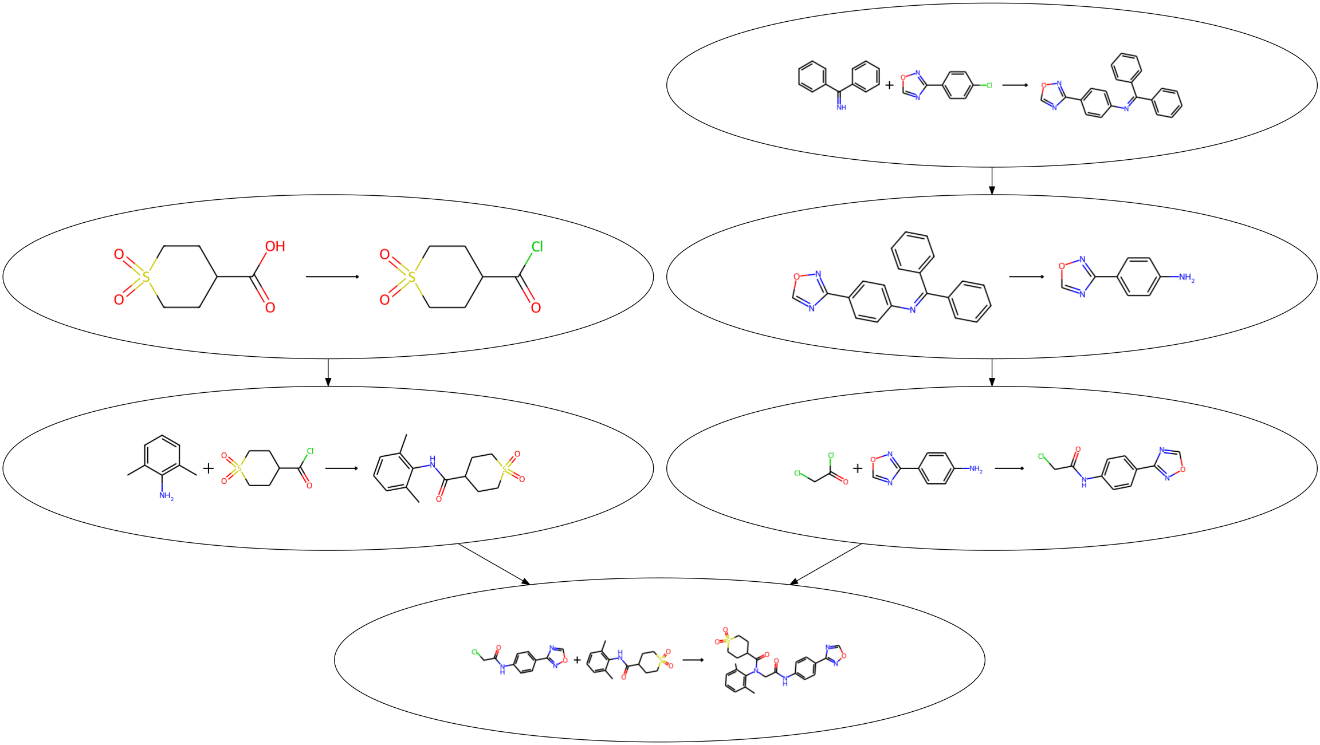


Figure S12: Most representative route for cluster 5.


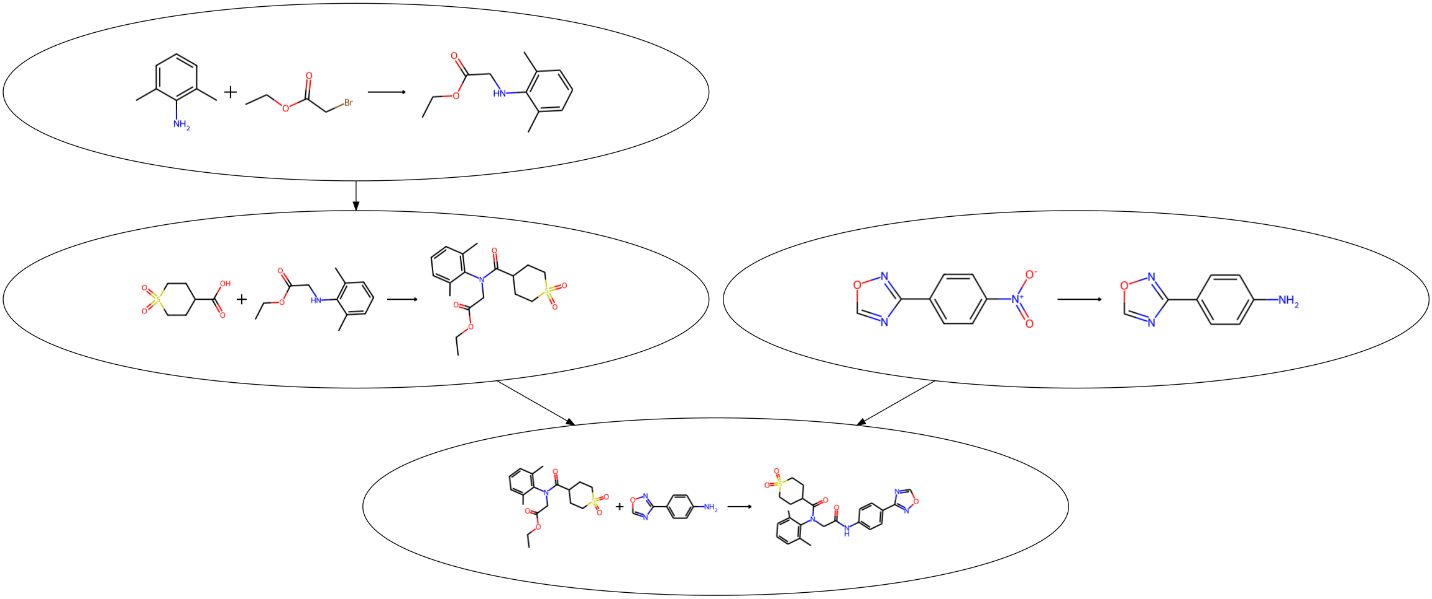


Figure S13: Most representative route for cluster -1 (route classified as "noise").
